# Detection and quantification of breast arterial calcifications on mammograms: a deep learning approach

**DOI:** 10.1007/s00330-023-09668-z

**Published:** 2023-05-09

**Authors:** Nazanin Mobini, Marina Codari, Francesca Riva, Maria Giovanna Ienco, Davide Capra, Andrea Cozzi, Serena Carriero, Diana Spinelli, Rubina Manuela Trimboli, Giuseppe Baselli, Francesco Sardanelli

**Affiliations:** 1 Department of Biomedical Sciences for Health, Università degli Studi di Milano, Milan, Italy; 2 Department of Radiology, Stanford University School of Medicine, Stanford, CA USA; 3 Department of Electronics, Information and Bioengineering, Politecnico di Milano, Milan, Italy; 4 Unit of Radiology, IRCCS Policlinico San Donato, San Donato Milanese, Milan, Italy; 5 Postgraduation School in Radiodiagnostics, Università degli Studi di Milano, Milan, Italy

**Keywords:** Cardiovascular diseases, Mammography, Risk factors, Vascular calcification, Artificial intelligence

## Abstract

**Objective:**

Breast arterial calcifications (BAC) are a sex-specific cardiovascular disease biomarker that might improve cardiovascular risk stratification in women. We implemented a deep convolutional neural network for automatic BAC detection and quantification.

**Methods:**

In this retrospective study, four readers labelled four-view mammograms as BAC positive (BAC+) or BAC negative (BAC−) at image level. Starting from a pretrained VGG16 model, we trained a convolutional neural network to discriminate BAC+ and BAC− mammograms. Accuracy, F1 score, and area under the receiver operating characteristic curve (AUC-ROC) were used to assess the diagnostic performance. Predictions of calcified areas were generated using the generalized gradient-weighted class activation mapping (Grad-CAM++) method, and their correlation with manual measurement of BAC length in a subset of cases was assessed using Spearman *ρ*.

**Results:**

A total 1493 women (198 BAC+) with a median age of 59 years (interquartile range 52–68) were included and partitioned in a training set of 410 cases (1640 views, 398 BAC+), validation set of 222 cases (888 views, 89 BAC+), and test set of 229 cases (916 views, 94 BAC+). The accuracy, F1 score, and AUC-ROC were 0.94, 0.86, and 0.98 in the training set; 0.96, 0.74, and 0.96 in the validation set; and 0.97, 0.80, and 0.95 in the test set, respectively. In 112 analyzed views, the Grad-CAM++ predictions displayed a strong correlation with BAC measured length (*ρ* = 0.88, *p* < 0.001).

**Conclusion:**

Our model showed promising performances in BAC detection and in quantification of BAC burden, showing a strong correlation with manual measurements.

**Clinical relevance statement:**

Integrating our model to clinical practice could improve BAC reporting without increasing clinical workload, facilitating large-scale studies on the impact of BAC as a biomarker of cardiovascular risk, raising awareness on women’s cardiovascular health, and leveraging mammographic screening.

**Key Points:**

*• We implemented a deep convolutional neural network (CNN) for BAC detection and quantification.*

*• Our CNN had an area under the receiving operator curve of 0.95 for BAC detection in the test set composed of 916 views, 94 of which were BAC+ .*

*• Furthermore, our CNN showed a strong correlation with manual BAC measurements (ρ = 0.88) in a set of 112 views.*

**Supplementary Information:**

The online version contains supplementary material available at 10.1007/s00330-023-09668-z.

## Background

Cardiovascular diseases (CVD) are the leading cause of death in the female population [1]. Although it is commonly assumed that males have a greater mortality rate from CVD [2], almost as many women as men die from heart disease yearly. Traditional approaches for cardiovascular risk assessment perform worse in women [3, 4], as up to 20% of women’s cardiovascular adverse events occur in the absence of traditional risk factors [5], and women are less likely to be prescribed CVD prevention therapy in primary care settings [6]. Hence, innovative imaging biomarkers that could improve cardiovascular risk stratification in women have been proposed over the last two decades [7].

**Fig. 1:**
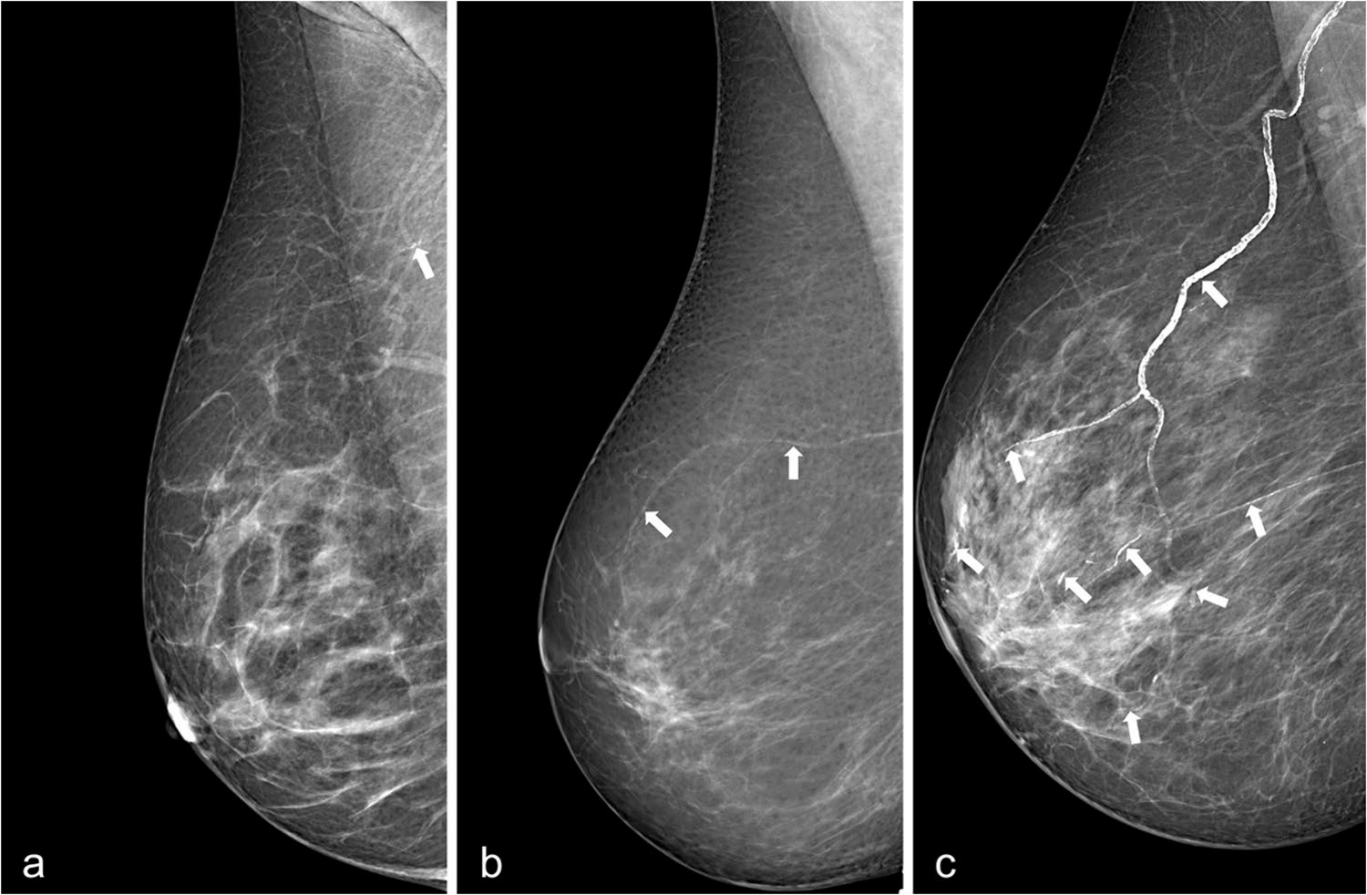
Examples of breast arterial calcifications on screening mammograms (white arrows). **a** Low, **b** mild, and **c** severe burden of BAC

In particular, breast arterial calcifications (BAC) have been suggested as a sex-specific predictor of cardiovascular risk [8–14]. BAC are a common incidental finding on mammograms, where they appear as parallel linear opacities within vessel walls (illustrated in Fig. 1) [8, 12]. Their approximate prevalence, although in a wide range, has been estimated around 13% [11, 13–16]. BAC presence has been associated with a 1.23 increased risk of CVD in postmenopausal women [14] and has higher diagnostic accuracy than other traditional cardiovascular risk factors in asymptomatic middle-aged women, especially under 60 years of age [10, 11, 13].

Considering the widespread diffusion of screening mammography [17, 18], systematic BAC assessment could provide a low-cost cardiovascular risk stratification in women without any additional tests. Although most radiologists are aware of the link between BAC and CVD, BAC reporting in routine mammography interpretation is scarce [19], being further prevented by the lack of standard BAC reporting guidelines and of reliable and quick methods for BAC detection and quantification [8, 15]. As BAC vary considerably in size, length, and density, several methods for BAC burden estimation have been proposed, either with manual semiquantitative scoring [15, 16] or with quantitative scoring based on automated segmentation by artificial neural networks [20, 21]. Despite promising results, these supervised algorithms still required time-consuming manual pixel-wise annotations in a large number of images for the training process. Conversely, deep learning (DL) algorithms and convolutional neural networks (CNN) trained for BAC detection using a simple dichotomic classification could provide higher robustness and lesser human image postprocessing workload [22, 23]. BAC positive (BAC+) and BAC negative (BAC−) annotation could therefore be adopted in place of a full manual segmentation of BAC, and throughout this work, we will refer to the former as “weak supervision” as opposed to the latter.

The objective of our study was to develop a weakly supervised deep CNN that can distinguish mammograms with and without BAC. Additionally, we aimed to obtain an estimate of the BAC burden as a by-product of our detection algorithm. To achieve this, we formulated the problem as a binary classification task and used an AI explainability algorithm to identify the approximate location of BAC, without relying on ground truth segmentation.

## Methods

### Patient enrolment and data collection

This retrospective study was approved by the local ethics committee (protocol code SenoRetro, approved on November 9, 2017, amended on May 12, 2021), and the need for informed consent was waived. We included a series of consecutive patients aged ≥ 45 years, who were referred to the IRCCS Policlinico San Donato between January and March 2018 to undergo spontaneous or organized population-based screening mammography.

All included examinations were bilateral mammograms with cranio-caudal (CC) and medio-lateral oblique (MLO) projections, acquired using full-field digital systems (Giotto IMAGE 3DL or Giotto TOMO series, IMS). Three readers (R.M.T., D.S., and S.C. with 10, 3, and 2 years of experience in breast imaging, respectively) reviewed the included mammograms to perform a patient-based classification as BAC+ or BAC− . BAC+ patients had at least one BAC detectable on a mammographic view, whereas all other patients were considered BAC − . A fourth reader (D.C. with 3 years of experience in breast imaging) then labelled each mammographic view of BAC+ patients as BAC+ or BAC− . All the labels were encoded in a database and served as the ground truth during training and testing of the BAC detection model.

### Clinical dataset preparation and pre-processing

To preserve the age distribution of the positives, BAC+ data was divided into four age classes using our population’s age quartiles as thresholds: first class, 45 years–Q1; second class, Q1–Q2; third class, Q2–Q3; and fourth class, Q3–maximum age of the participants (see “Results” for details). Then, we performed a stratified split of the BAC+ dataset into three subsets within the classes to preserve the BAC+ age distribution: 70% of the random shuffled positive cases entered the training subset, 15% entered the validation subset to tune model hyperparameters based on the highest precision-recall curve (AUC-PR), and the remaining 15% entered the test subset to evaluate the performance of the final optimized CNN. Subsequently, the whole BAC− dataset was randomly partitioned into training, validation, and test sets containing 70%, 15%, and 15% of the negative cases, respectively. The relevant BAC+ and BAC− splits were then consolidated to complete the three subsets. To account for class imbalance during model training [24, 25], the majority class (BAC−) in the training subset was randomly under-sampled to reach a BAC+ prevalence of 30% at patient level. The validation and test sets remained intact to mirror the real BAC prevalence. To eliminate any bias that may happen by allocating different views of a single case into different subsets, data splitting at patient level preserved all the mammogram views of each case in the same subset.

A data pre-processing step was also required to exclude non-tissue areas. Using histogram analysis following Otsu’s method, we successfully extracted the tissue regions from the dark background pixels [26, 27]. Then, after defining the smallest rectangular area surrounding the breast tissue, the cropped images were scaled to a fixed-size matrix that would define the size of the input layer of the CNN (Fig. S1). Pixels belonging to the breast region were normalized to improve the convergence of training, thus accounting for the high variability of mammogram pixel intensities caused by acquisition and biological factors like technical differences between mammography units and tissue density.

### Neural network architecture and implementation

**Fig. 2:**
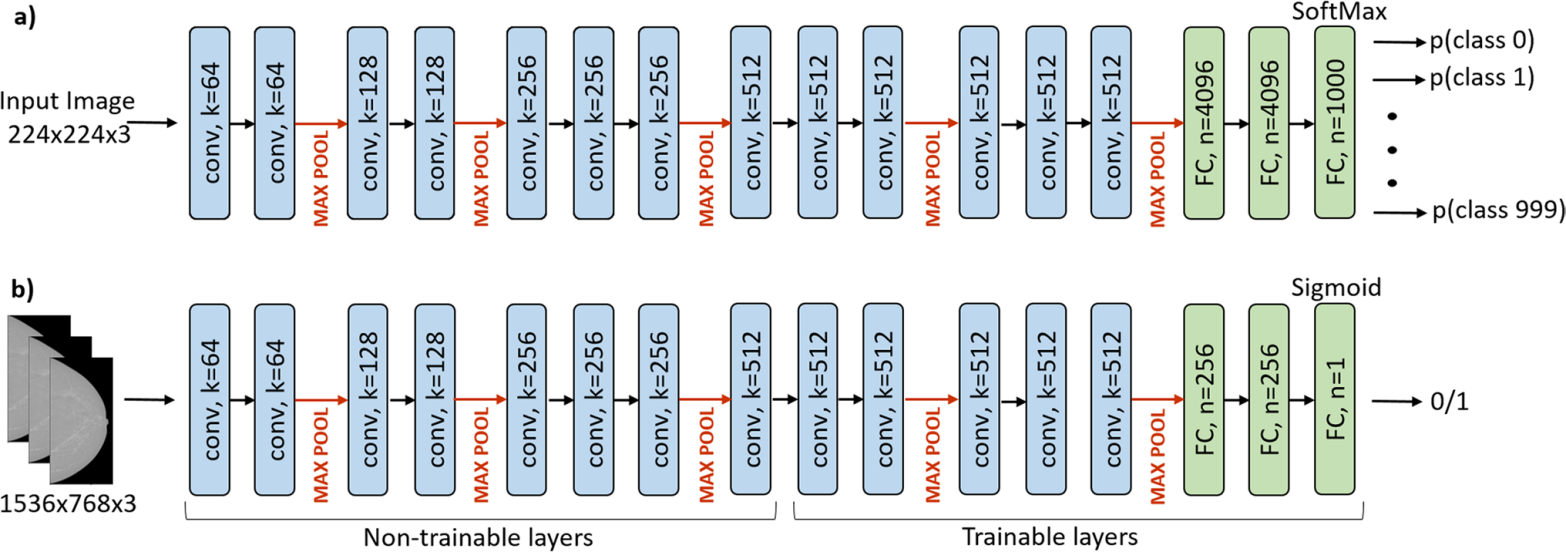
General VGG16 architecture consisting of 13 convolutional layers (kernel 3 × 3, depth *k*), 5 pooling layers (non-trainable), and 2 fully connected (FC, *n*: number of neurons) layers followed by a Softmax activation function to solve the multiclass classification problem (**a**), and the final CNN for automated binary BAC detection where the “non-trainable layers” exploited VGG16 transfer learning (**b**). Rectified linear unit (ReLU) activation functions (in model a) and leaky ReLUs (in model b) following each convolutional kernel are not shown

We implemented a BAC detection model using a deep transfer learning strategy [28] based on the 16-layer pretrained Visual Geometry Group (VGG16) image classification model with modifiable connection weights [29]. We replaced the last dense layer with two fully connected layers (256 channels each) including leaky rectified linear unit activation functions (*α* = 0.3) trained from scratch, and a sigmoid activation function as final output layer, as appropriate for our binary classification problem (presence or absence of BAC). Next, we optimized the number of the initial convolutional layers to be fixed as “non-trainable layers” and of the later ones to be fine-tuned on the new binary classification. This was done by trial and error, each time training the modified CNN and assessing its performance on the validation set. The best-performing structure was found to be that with five fine-tuning layers. Figure 1 summarizes the complete architecture of the proposed CNN, which was developed using Python V3.8.11 on a system with NVIDIA GeForce RTX 3080, 10 GB VRAM. VGG16 input structure constrained a fixed dimension of red–green–blue color coding (Fig. 2a); hence, gray-level mammograms were resampled to fixed-size 1536 × 768 images and input three times in parallel (Fig. 2b). Our model elaborated each mammographic view independently.

We applied online data augmentation during training, including random rotations, width/height shift, horizontal/vertical flip, and zoom, as well as random Gaussian and salt–pepper noise addition to learn more robust features. During training, the Adam optimizer [30] was applied to minimize the binary cross-entropy loss function. Class-balanced re-weighting strategy was also utilized to deal with the imbalanced dataset at algorithm level which automatically altered the loss inversely proportional to the class frequency, thereby assigning higher costs to the minority BAC + class. Learning rate was initially set to 10^−6^ and adjusted over the epochs using cosine annealing scheduler [31]. Due to the highly imbalanced dataset, the area under the PR curve was monitored and the parameters related to the maximum quantity provided the best model configuration at the end of each epoch. The number of epochs and batch size were selected to be 25 and 8 images, respectively. Dropout regularization was set to 0.3 for each dense layer. The loss curves are represented in figure S2.

Finally, visual explanations of the proposed CNN were generated using the generalized gradient-weighted class activation mapping (Grad-CAM++) method after the deepest convolutional layer [32, 33], providing heatmaps highlighting the pixels that were significant for predictions. Simple binarization thresholding of the heatmaps in positive predictions enabled us to delineate an estimated BAC region from the total tissue.

The time required for automatic mammogram classification and generation of Grad-CAM++ heatmaps was recorded and reported as average image elaboration time.

### Quantification

We assessed the correlation of the estimated BAC region area delineated on the Grad-CAM++ in a subset of MLO views with manual measurements of calcified segment lengths obtained from a previously published study [15]. The BAC area was calculated as follows:

$${\mathrm{BAC}}_{\mathrm{area}}= P{\sum }_{i=1}^{n}{1}_{G\left(i\right)>Th}$$

where $P$ is the pixel size, $n$ the total number of pixels in the image, and $G\left(i\right)$ the Grad-CAM++ heatmap value at pixel *i*^th^. $Th$ or the best binarization threshold was set to 0.3 by trial and error.

### Statistical analysis

The Kolmogorov–Smirnov test was used to assess the normality of the continuous variables’ distributions; normal variables were reported as mean ± standard deviation (SD), whereas non-normal variables were reported as median and interquartile range (IQR). The Mann–Whitney *U* test was performed to compare the age distributions in the BAC+ and BAC− groups; *p* values less than 0.05 were considered statistically significant [34].

The overall diagnostic performance of the proposed CNN model was evaluated against the ground truth labels provided by the readers, using the following metrics: accuracy, precision, recall, F1 score, and area under the receiver operating characteristic curve (AUC-ROC). Correlations were appraised by Pearson *r* or Spearman *ρ* as appropriate, and the resulting coefficients were interpreted according to Evans [35].

## Results

A total of 1557 patients underwent screening mammography at our institute between January and March 2018. After excluding patients younger than 45 years of age, 1493 women with a median age of 59 years (IQR 52–68) were finally included, for a total of 5972 mammographic views. BAC were present in 194 of 1493 women (13.0%) and 581 of 5972 views (9.7%), respectively. The prevalence of BAC increased with age, from 6.3% in the first age class (45–60 years) to 11.6% in the second age class (61–70 years), 34.3% in the third age class (71–73 years), and 38.2% in the fourth age class (74–87 years). The 194 BAC+ women had a significantly higher median age (70.5 years, IQR 60–73) than the 1299 BAC− women (median 57 years, IQR 52–65, *p* < 0.001).

Table 1 reports training, validation, and test set composition. Following data partitioning, 1042 women (4168 mammograms) were assigned for training, 222 (888 mammograms) for validating, and 229 (916 mammograms) for testing, each containing 398, 89, and 94 BAC+ views, respectively. To reduce class imbalance during model training, we artificially increased the prevalence of BAC+ patients to around 30% in the training set by randomly undersampling BAC− mammograms from those assigned to the training dataset, reaching 1640 images. Eventually, image-level BAC prevalence was lower, given that not all mammographic views of BAC+ patients showed BAC. BAC prevalence in the validation and test sets was left unchanged.

**Table 1:** Training, validation, and test set composition

	Training set	Validation set	Test set
BAC+ , *n* (%)	398 (24.27)	89 (10.02)	94 (10.26)
BAC− , *n* (%)	1242 (75.73)	799 (89.98)	822 (89.74)
Total images	1640	888	916

**Fig. 3:**
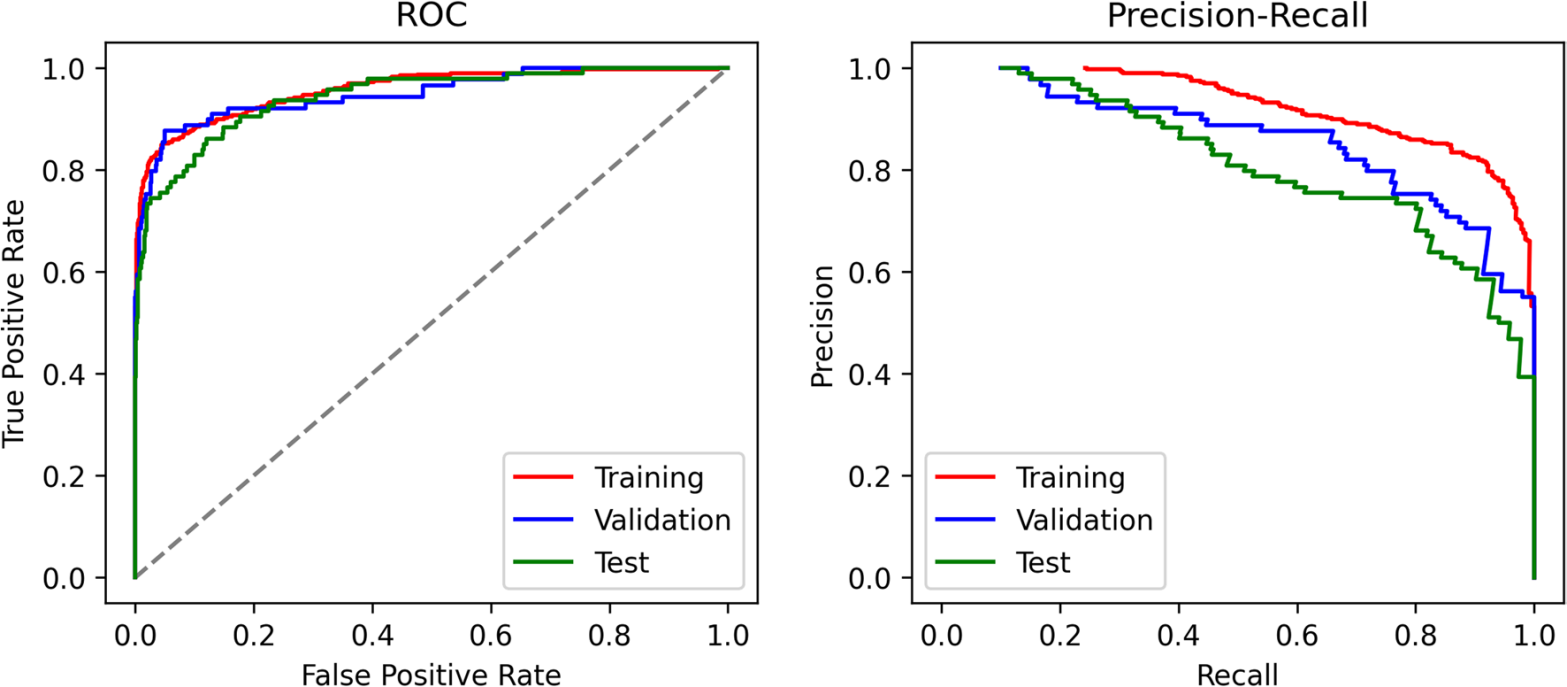
ROC and PR curves of training (red line), validation (blue line), and test (green line) subsets

Table 2 represents the overall corresponding image-level performances of the proposed CNN model in detecting the presence or absence of BAC in the subsets. Training was performed at image level and optimized based on the highest AUC-PR. In the independent test set, the best-trained CNN achieved a 0.95 accuracy, a 0.76 F1 score, and a 0.94 AUC-ROC, highlighting good overall performances in BAC detection. The ROC and PR curves of all subsets are presented in Fig. 3.

**Table 2:** Diagnostic performance of the model in detecting BAC on mammograms

	TN	TP	FN	FP	Accuracy	Balanced accuracy	Precision	Recall	F1 score	AUC-ROC	AUC-PR
Training	1222	312	86	20	0.93	0.88	0.94	0.78	0.85	0.96	0.93
Validation	787	64	25	12	0.96	0.85	0.84	0.72	0.78	0.95	0.86
Test	803	69	25	19	0.95	0.86	0.78	0.73	0.76	0.94	0.81

*TN* true negative, *TP* true positive, *FN* false negative, *FP* false positive, *AUC-ROC* area under the receiver operating characteristic curve, *AUC-PR* area under the precision-recall curve

**Fig. 4:**
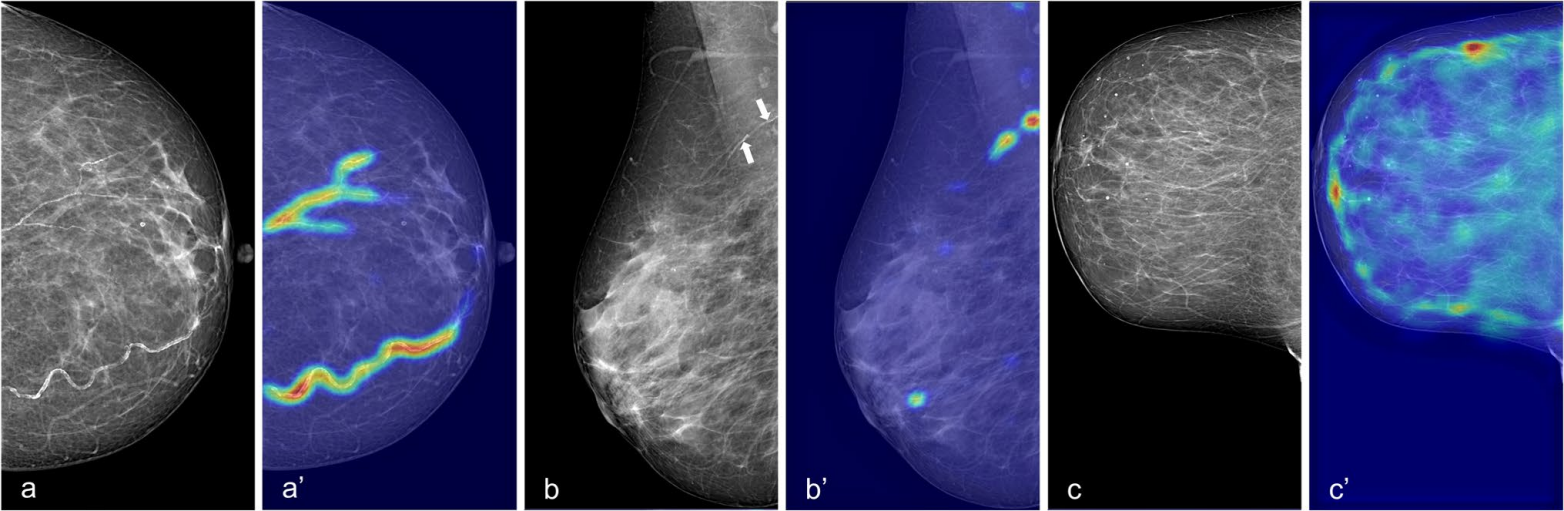
Visual explanations (Grad-CAM++ heatmaps) of the automatic detection results by the proposed model. **a**, **a′** True-positive case with a high burden of BAC in multiple vessels; **b**, **b′** true-positive case with small BAC (arrows); **c**, **c′** true-negative case with confounding factors, i.e. various benign calcifications (none of the structures colored on the heatmap reaches the threshold for being finally detected as BAC)

**Fig. 5:**
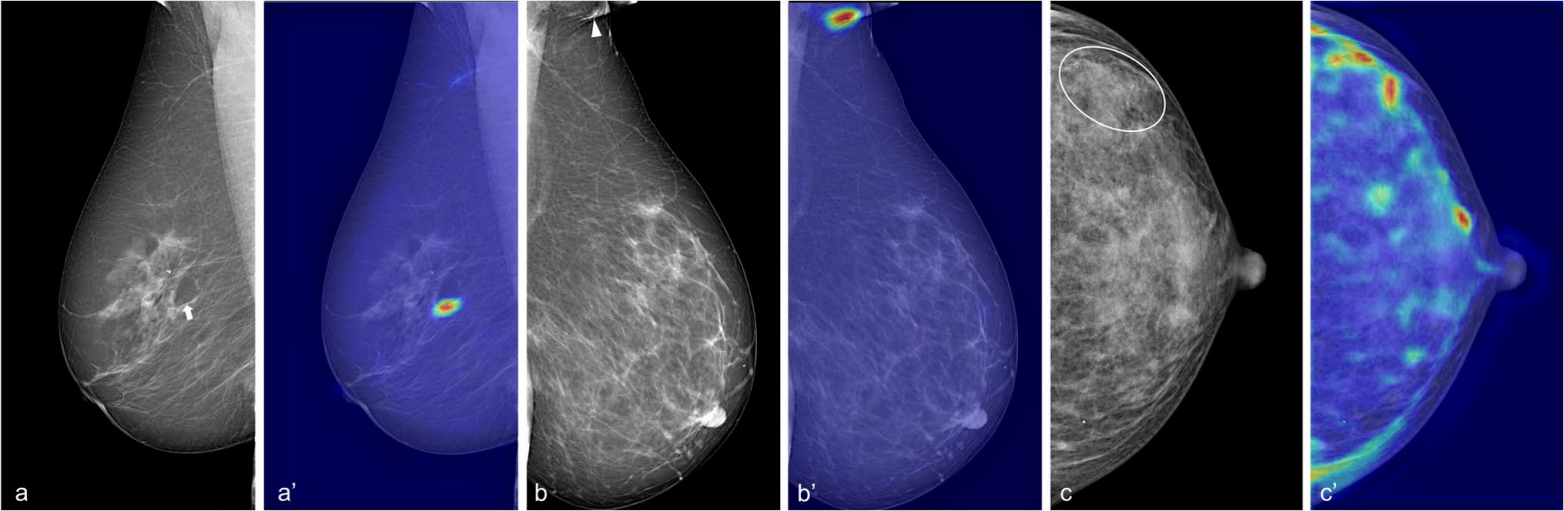
Examples of misclassification. **a**, **a′** False-positive case with small calcifications within a Cooper’s ligament mistaken as BAC (arrow), **b**, **b′** false-positive case with skinfold including cutaneous calcifications mislabelled as BAC (arrowhead), **c**, **c′** false-negative case with small BAC concealed under dense tissue (circle)

Figure 4 shows the performance of our CNN model through Grad-CAM++ heatmaps. In true-positive detections, BAC are accurately localized also when multiple incidences of BAC are present in the same view (Fig. 4a, a′). Furthermore, our CNN demonstrated to be capable of detecting even small BAC occurrences (Fig. 4b, b′). Conversely, Grad-CAM++ heatmaps of true-negative predictions emphasize BAC-like structures in the whole breast without reaching the threshold for BAC+ classification (Fig. 4c, c′) and without being confounded by typically benign rounded calcifications. Examples of wrong detection are reported in Fig. 5. The average image elaboration time, including automatic BAC detection and Grad-CAM++ generation, was 0.80 ± 0.07 s.

**Fig. 6:**
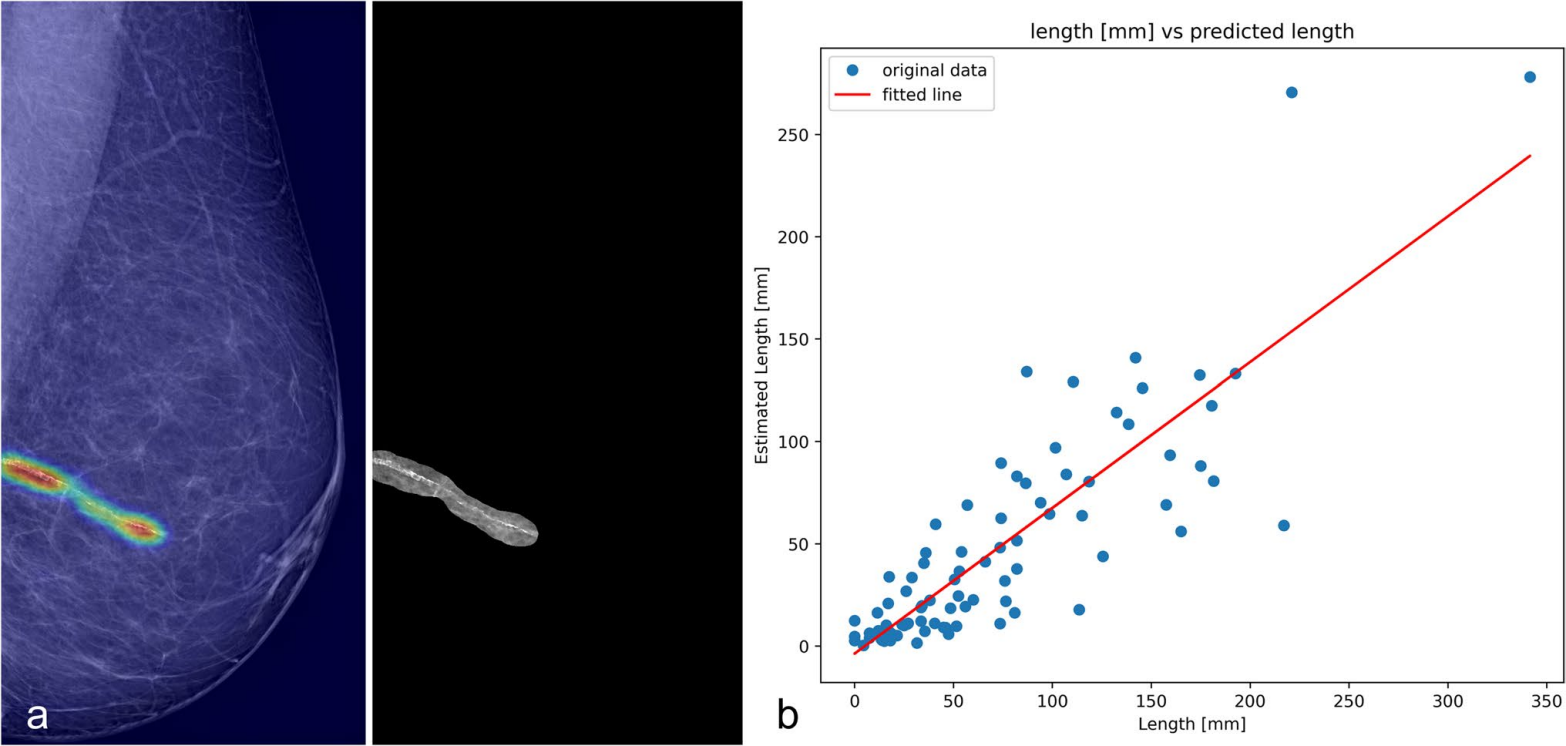
**a** Automatic segmentation of a BAC by thresholding the Grad-CAM++ heatmap of a mammogram with moderate burden of BAC (length 41 mm). **b** Scatterplot of the estimated area (*y*-axis) compared to the manually measured length (*x*-axis) for all 56 women in the subgroup (112 views)

A preliminary quantitative evaluation was performed on a subgroup of 57 patients with previous manual BAC length measurements. One patient had a discordant assessment of her BAC status between assigned label and BAC length measurement and was hence discarded. The analysis was therefore performed on MLO views of 56 BAC+ women aged 49–82 years. In total, 112 MLO views were analyzed, and presence of BAC was reported in 95 of them. Automatic BAC burden estimation was performed by Grad-CAM++ heatmaps thresholding as depicted in Fig. 6a. The automatically detected BAC area showed a strong correlation with the manually measured length (Spearman *ρ* = 0.88, *p* < 0.001) (Fig. 6b).

## Discussion

We implemented a CNN for the automatic detection of BAC on mammograms. Our model showed good performances in BAC detection, with an AUC-ROC of 0.95 in the test set, and it proved capable of estimating BAC area with a correlation of 0.88 with manual measurements. The application time of our model was less than a second for each image, a time suitable for a swift integration in everyday clinical practice.

In the framework of the research effort aiming to reduce the gender gap in CVD prevention and cardiovascular risk assessment [36], BAC stand out as a beneficial and low-cost biomarker of cardiovascular risk that can be easily obtained from the already established mammographic screening practice [37]. Nonetheless, BAC presence is seldom reported during mammography interpretation [19]: this can be ascribed both to the primary focus on cancer detection that clinicians keep in the context of mammographic screening and to the lack of fast, automated, and reliable tools for BAC detection and quantification. Therefore, automatic tools for BAC detection and quantification could overcome this issue without increasing the radiologists’ workload.

A previous experience in BAC semiautomatic detection and quantification demonstrated that human detection is the main source of variability in developing an automated tool [38]. Therefore, we chose to address the classification problem by training a weakly supervised CNN, which may allow partially overcoming the intra- and inter-reader variability. Our CNN was trained using image-level labels in order to obtain as by-product then pixel-wise detection of BAC on mammograms [39]. This strategy allowed us to reach high performances with an accuracy of 0.95, a recall (i.e. sensitivity) of 0.73, a precision (i.e. positive predictive value) of 0.78, and an AUC-ROC of 0.94 in the independent test set, which consisted of 916 images. Furthermore, our model proved to be capable of estimating BAC area with a strong correlation (*ρ* = 0.88) with manual annotation in a subset of 56 positive cases.

Our performances are similar to those reported by previous studies: Khan and Masala [40] recently published a study on BAC detection using transfer learning, comparing the results obtained from different deep learning architectures trained on a small population of just 104 mammograms from 26 patients. They reported an accuracy of 0.96 of VGG19, marginally lower than that yielded by deeper CNNs such as ResNet50 or DenseNet-121, which showed an accuracy of 0.97 and 0.98, respectively. In 2017, Wang et al [21] developed a CNN for BAC detection using the mammograms of 210 women, 146 BAC+ and 64 BAC− , demonstrating a detection rate comparable to that of human readers, and a very strong correlation between the automatically estimated BAC area and the ground truth (Pearson coefficient 0.94). In 2021, Guo et al [20] trained a Simple Context U-Net capable of segmenting BAC with an *R*^2^ correlation > 0.95 with ground truth. The estimated area using this model was strongly correlated with calcification volume (*R*^2^ = 0.84) and calcification mass (*R*^2^ = 0.87) on breast computed tomography. However, some notable advantages of our model over these previously developed tools are worth noting. First, we did not input any information regarding BAC quantity for CNN training, whereas Guo and Wang’s works relied on manual, pixel-by-pixel BAC annotations as ground truth [20, 21]. Our weakly supervised approach yielded a twofold benefit: a considerable facilitation in the dataset formation (as our readers only had to classify each image either as BAC+ or BAC−) and a sizable computational efficiency, given that we obtained good estimations of BAC burden as a by-product of BAC detection using a relatively simple CNN, with fast processing times (around 1 s for each image). Furthermore, differently from previous works, we tested our model on an independent test set which reflected real-world BAC prevalence (around 12%), whereas the datasets employed in other works [20, 21] included a majority of BAC+ patients, which might have led to model overfitting [41]. Instead, we chose to artificially augment BAC prevalence to 30% only in the training set, in order to select the best-performing hyperparameters for BAC detection, and then reverted to a 12% prevalence for validation and testing. Therefore, as we already tested the CNN on a realistic and imbalanced set, we hypothesize that our model’s performances will be stable and robust in the upcoming external validation, where BAC are the minority class.

A visual examination of the wrong predictions by our model showed that the majority of false positives were due to small calcifications that mimicked BAC usual appearance, i.e. lined-up, punctuated calcifications often within linear formations such as skin folds or Cooper’s ligaments (Fig. 5a, b). Conversely, false negatives occurred in situations where BAC detection could be difficult also for trained human readers, such as BAC in dense breasts (Fig. 5c) or very faint BAC. Of note, the latter could perhaps be of lower clinical value for CVD risk prediction.

Our work presents some limitations. First, the model was trained and tested on a consecutive series of women from a single institution studied using two mammographic units from a single manufacturer. Even though our dataset consisted of over 1400 patients and we allotted 15% of the dataset for independent testing, an external validation of our model on different machines is warranted. Second, the correlation coefficient of BAC burden estimation with manual measurement in our work (0.88) was marginally lower than those reported in previous studies (0.95 [20] and 0.94 [21]). However, we must note that differently from previous studies, we did not train our model using manual segmentations as ground truth, and that extremely precise BAC segmentation may not be necessary from a clinical point of view. Indeed, according to the most recent meta-analysis on the association between BAC and CVD [42], only moderate and severe BAC (i.e. extensive calcifications on one or more vessels, clouding the vessels’ lumen and involving notable portions of their length—see Fig. 2a) were associated with coronary artery disease. Therefore, our model would still allow identifying women at higher CVD risk, albeit with a less precise BAC segmentation. Third, we performed a stratified split of BAC+ cases into training, validation, and test sets to preserve the BAC age distribution and avoid any age-related potential bias. However, this procedure might have introduced some degree of sampling bias, considering the age constrains in the randomization. Finally, we did not perform any experimental comparison between the performances of our model and that obtainable with other available CNN architectures, such as ResNet 50 or DenseNet. However, such comparison was beyond the aims of the present work.

In conclusion, we developed a CNN that can detect BAC with good performance (AUC-ROC of 0.94 in the test set) and can also output a segmentation of BAC with a very strong correlation with manual measurements (*ρ* = 0.88). The integration of our model to clinical practice could improve BAC reporting without increasing clinical workload, potentially facilitating large-scale studies on the impact of BAC use as a biomarker to consistently guide cardiovascular risk assessment and management, ultimately contributing to raise awareness on women’s cardiovascular health in the context of mammographic screening practice.

### Supplementary Information

Below is the link to the electronic supplementary material.

Supplementary file1 (PDF 242 KB)

## Abbreviations

AUC-PR: Area under the precision-recall curve
AUC-ROC: Area under the receiver operating characteristic curve
BAC: Breast arterial calcifications
CC: Cranio-caudal
CNN: Convolutional neural network
CVD: Cardiovascular diseases
DL: Deep learning
Grad-CAM++: Generalized gradient-weighted class activation mapping
IQR: Interquartile range
MLO: Medio-lateral oblique
SD: Standard deviation
VGG16: Visual Geometry Group 16

## Declarations

### Guarantor

The scientific guarantor of this publication is Professor Francesco Sardanelli.

### Statistics and biometry

One of the authors has significant statistical expertise.

### Study subjects or cohorts overlap

Four hundred eight study subjects have been previously included in a paper (Trimboli et al, QIMS 2021, https://doi.org/10.21037/qims-20-560) that was focused on manual segmentation of breast arterial calcifications and did not share any study aim or methodological aspects with the present work.

### Methodology

• retrospective

• experimental

• performed at one institution
