# Supplementary material for: Detection and quantification of breast arterial calcifications on mammograms: a deep learning approach
Source: Eur Radiol. 2023 May 9;33(10):6746–55. doi: 10.1007/s00330-023-09668-z (PMC10511622; doi:10.1007/s00330-023-09668-z)

Supplementary figures

Fig. S1 Overall workflow of the pre-processing step to exclude non-tissue areas. a) a sample mammogram with a matrix size of 3580x2812, b) the bimodal histogram (low intensities referring to the background, high intensities referring to the tissue) and the calculated Otsu's threshold, c) the smallest rectangular contour surrounding the largest over-threshold area as the breast tissue, and d) final cropped and padded image to a fixed-size matrix of 1536x768

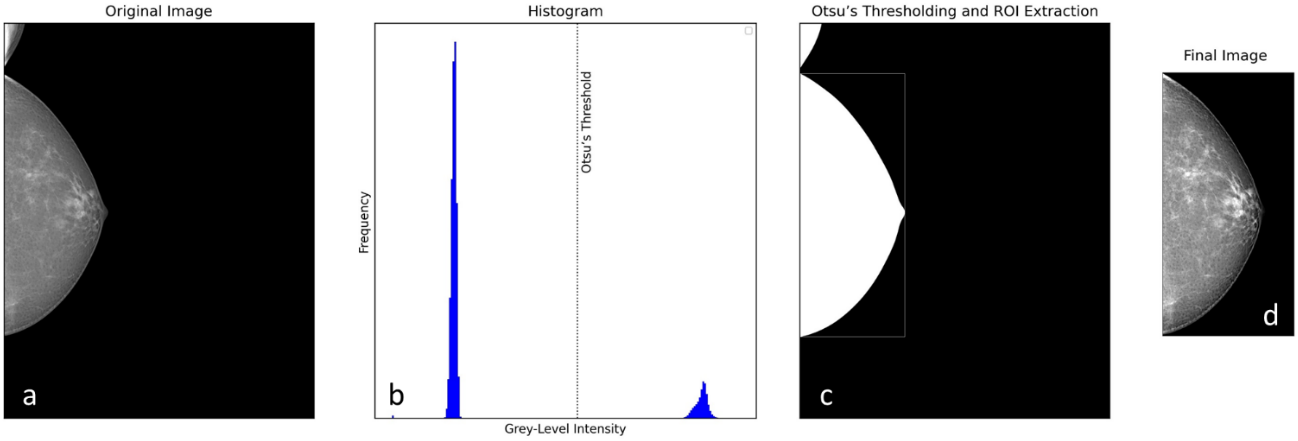

Fig. S2 Model loss curves over the epoch numbers.

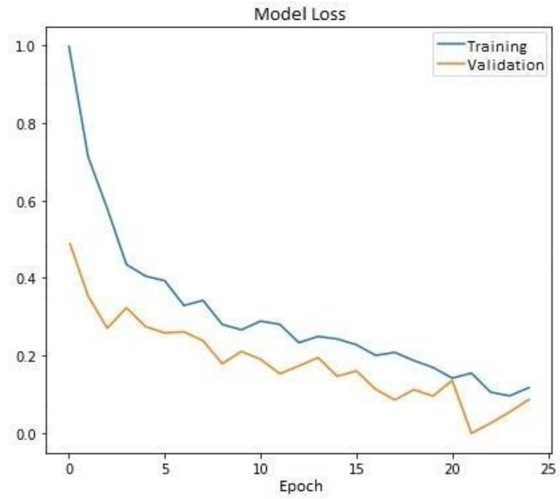

Supplement: Supplementary file 1 — Supplementary file1 (PDF 242 KB) [file 330_2023_9668_MOESM1_ESM.pdf]
